# MicroRNA-30e reduces cell growth and enhances drug sensitivity to gefitinib in lung carcinoma

**DOI:** 10.18632/oncotarget.13944

**Published:** 2016-12-15

**Authors:** Zhi-Qiang Ning, Hai-lin Lu, Chao Chen, Lin Wang, Wei Cai, Yan Li, Ting-hua Cao, Jing Zhu, Yong-Qian Shu, Hua Shen

**Affiliations:** ^1^ Department of Oncology, The First People's Hospital of Wujiang District, Suzhou, 215200, China; ^2^ Institute of Medcine, University of Zhengzhou, Henan Province, 450000, China; ^3^ Department of Oncology, The First Affiliated Hospital of Nanjing Medical University, Nanjing, Jiangsu, 210029, China; ^4^ Collaborative Innovation Center for Cancer Medicine, Jiangsu Key Lab of Cancer Biomarkers, Prevention and Treatment, Nanjing Medical University, Nanjing 210029, Jiangsu Province, China

**Keywords:** miRNA-30e, chemosensitivity, gefitinib, HOXA1, lung cancer

## Abstract

MicroRNAs (miRNAs) play critical roles in variousbiological processes,including malignancy. Here, we demonstrated that miR-30e levels were markedly reduced in human lung carcinoma specimens in comparisonwith adjacent normal tissues. In addition, miR-30eamounts were starkly lower in the resistant PC9/gefitinib (PC9G) cancer cells compared with PC9 cells. Meanwhile, miR-30eoverexpression inPC9G cells resulted in reduced cell proliferation and migration,reversing drug resistance to gefitinib.Conversely,miR-30e silencing in PC9 cells increased proliferation as well as migration, and conferred resistance to gefitinib.Moreover, HOXA1, which was identified asa new miR-30etarget, plays important roles in regulating cell fate, early developmental patterns and organogenesis.Importantly, miR-30ealso inhibited PC9G growth *in vivo*. Taken together, these findings demonstrated that miR-30eshould be considered a tumor suppressor miRNA, which could be used in treatinghuman lung cancer.

## INTRODUCTION

Lung carcinoma is the top killer among cancers, claiming 1.4 million lives in the world yearly. Non-small-cell lungcarcinoma (NSCLC) is found in about 80% of primary lungcancer patients, who are usually diagnosed in advanced stage despite current efforts and improvements aimed at early diagnosis [1]. Although a variety of therapeutic options are available for lung cancer patients, e.g. surgery, chemotherapy and radiotherapy, five-year survival ratesremain critically low. In patients with epidermal growth factor receptor (EGFR) activatingmutation, treatment with EGFR tyrosine kinase inhibitors (TKIs), e.g. gefitinib, shows high efficacy [2]. However, acquired TKI resistance hampers the use of such molecules. Increasing evidence suggests that miRNAs may significantly affect the development and chemoresistance of lung cancer [3–6].

MiRNAs are endogenous single-stranded non-coding RNAsassociated with various types of cancer [7]. They have essential functions in gene regulation, andeffect many important pathophysiological processes such as differentiation, development and tumorigenesis [8–10]. MiRNAs mainly bind to the 3′-untranslated region (3′-UTR) of mRNA molecules, suppressing protein synthesis through mRNA degradation or translational repression [11]. MiR-30ewas described as a tumor suppressor gene in various cancers [12–14]. Here, we found that overexpressionof miR-30einlung cancer cells resulted inreduced cell proliferation and migration, reversing drug resistance to gefitinib. Known miR-30e targets include Bmi1, P4HA1, BCR-ABL and UBC9 [12–15]. However, how miR-30eregulates lung cancer tumorigenesisremains unclear.

HOX genes belong to a highly conserved subgroup of theHomeobox superfamily which characteristically encodea 60-amino acid long DNA-binding motif. HOX genes play critical roles in regulating cell fate as well as early developmental events and organogenesis [16–18]. Alterations in HOX genes are also associated with multiple cancers in humans, e.g. lung, breast, and hematological cancers [19–21]. Here, we demonstrated that miR-30e targetedHOXA1, whose expression was reduced in miR-30e treated cells and conversely enhanced after miR-30e inhibition.

It has been reported that miR-30e may play important roles in cancerby affecting different signaling pathways. The current data revealed that miR-30eamounts were reduced in PC9Gcells in comparison with PC9 cells. We further characterized miR-30e and explore its molecular mechanisms in lung cancer. Interestingly, ectopic expression of miR-30e resulted reduced cell proliferation andmigration, withinduced apoptosisin lung cancer cells by suppressing the key target HOXA1. In addition, miR-30e rendered PC9G cells more sensitive to gefitinib *in vitro* and *in vivo*. These findings revealed a novel mechanism for miR-30e, indicating that this miRNA could be further assessed for the development of lung cancer therapeutics.

## RESULTS

### MicroRNA-30e is markedly downregulated in lung carcinoma

**Figure 1:**
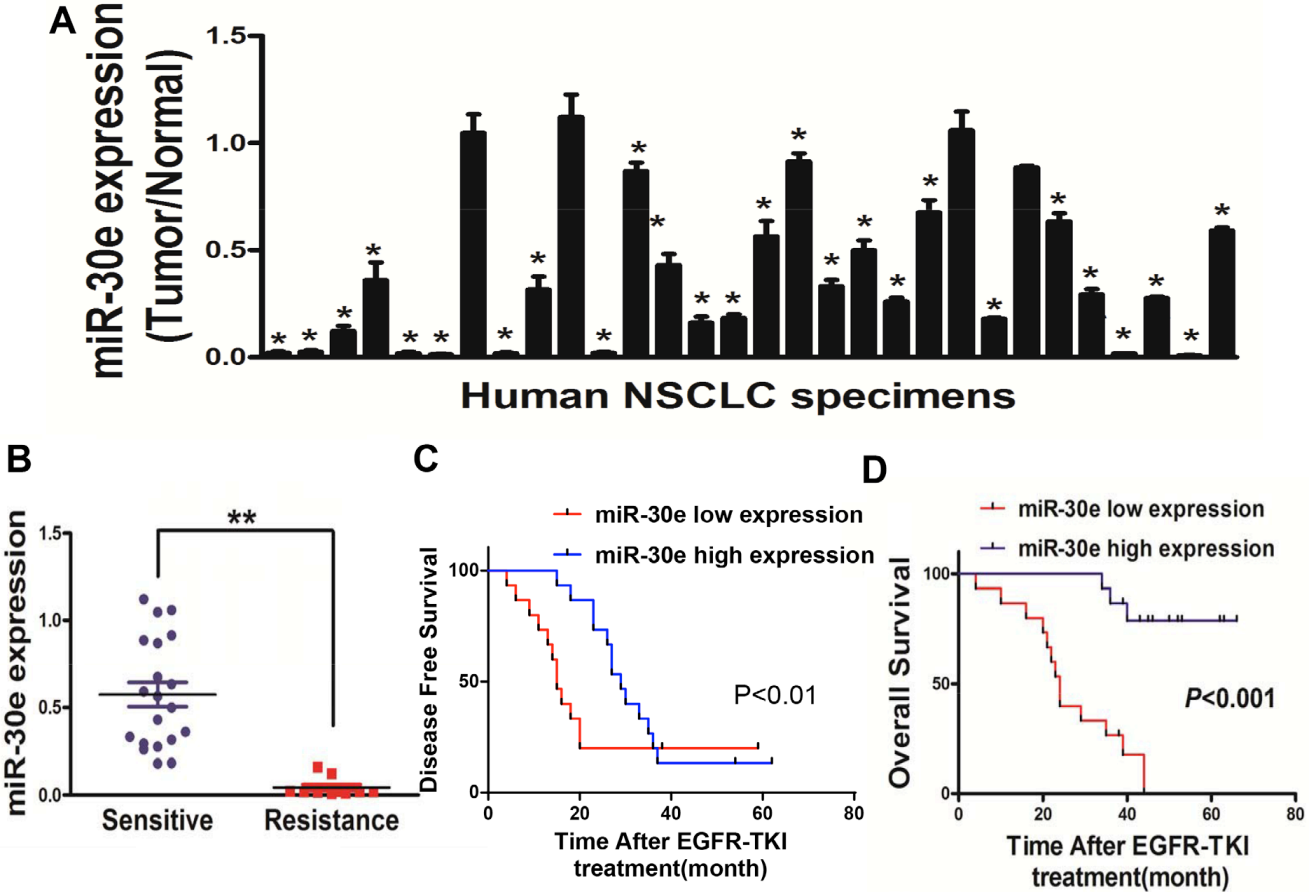
MiR-30e expression is markedlyreduced in lung cancer (**A**) Relative miR-30e amounts analyzed by qRT-PCR in 30 human lung cancer tissues alongside adjacent non-cancerous specimens, with U6 employed for normalization. (**B**) MicroRNA-30e amounts werereduced in lung cancer patients resistant to gefitinib. (**C**, **D**) Kaplan-Meier curves showingdisease-free-and overallsurvival based on miR-30e amounts. High and low miR-30e amounts were defined based on the 50th percentile value. Data represent mean ± SD of three replicates. * and ** indicate significant differences at *P* < 0.05 and *P* < 0.01, respectively.

To assessthe role of miR-30e in lung cancer, miR-30e amounts were assessed in 30 lung cancer tissues with the corresponding adjacent normal tissuespecimens. RT-qPCR showed that miR-30e was significant downregulated in lung cancer tissues (Figure 1A). Then, human lung cancer specimens were divided into 2 groups based on sensitivity of gefitinib; interestingly, loweramounts of miR-30ewere obtained in lung cancer patients with gefitinib resistance (Figure 1B). To evaluate the effect of miR-30e on NSCLC patientprognosis after treatment with EGFR-TKI, the Kaplan-Meier method and log-rank test were used to examine normalized miR-30e levelsand Disease free survival (DFS). Individuals displaying low miR-30e levels showed reduced DFS as well asoverall survival (OS) compared with thosedisplaying elevated miR-30e amounts (Figure 1C, 1D). Thesefindings suggested that loss of miR-30e may be associated with lung cancer disease progression, and should be considered a potential new biomarker for predicting poor prognosis in NSCLC.

### PC9G cells shows higher activity of proliferation and migration, and lower apoptosis rates compared with PC9 cells

**Figure 2:**
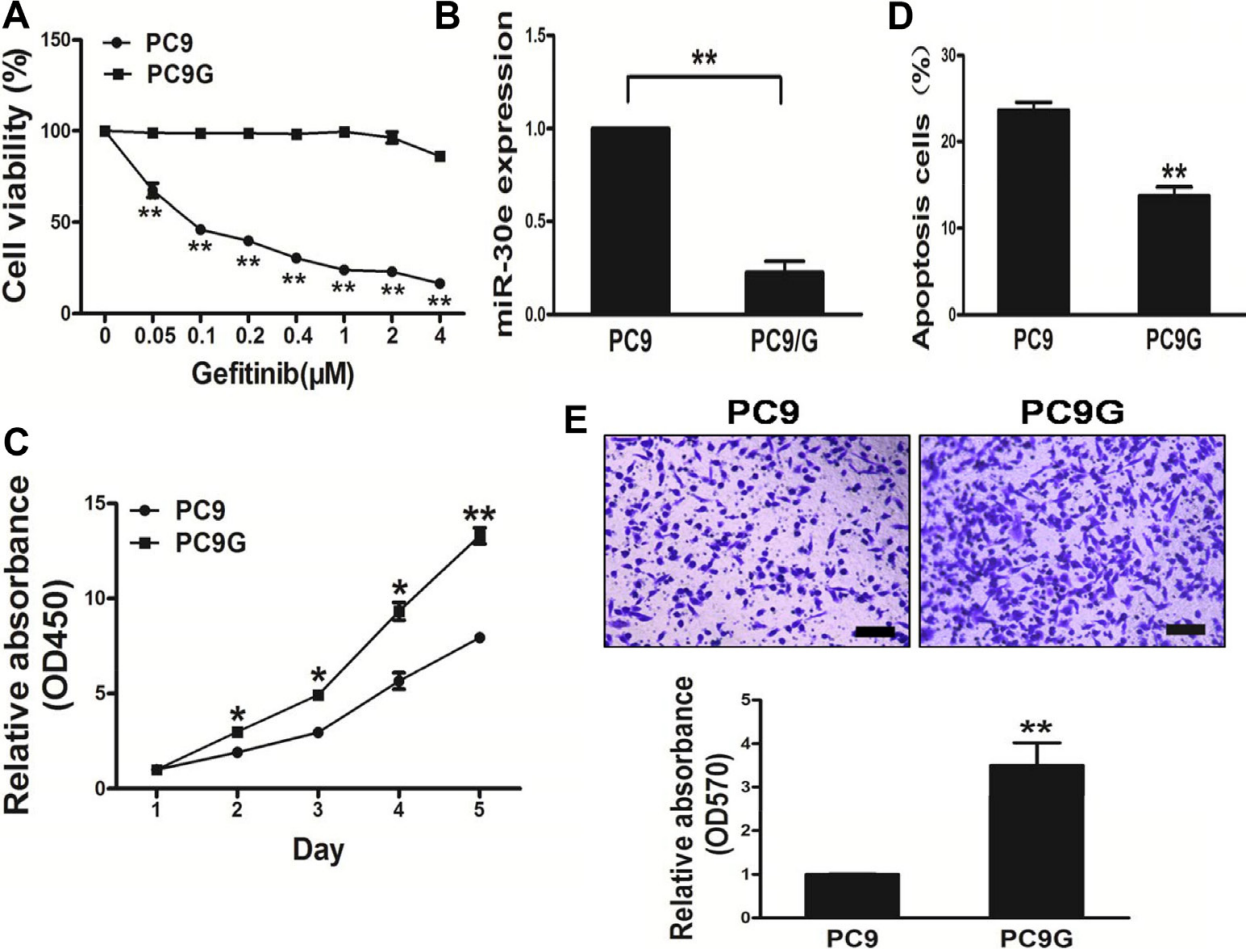
PC9Gcells show enhanced proliferation andmigration, and reduced apoptosis compared with PC9 cells (**A**) Compared with PC9 cells, PC9G cells displayed reduced sensitivity to gefitinib. (**B**) MicroRNA-30e expression in PC9 and PC9G cells. (**C**) The CCK8 assay was used to determine cell viability of PC9 and PC9Gat various time points. (**D**) Transwell migration assays was conducted for respective cells. (**E**) Apoptosis assay was performed for PC9 and PC9G cells. Data are mean ± SDof 3 replicate experiments. * and ** indicate significant differences at *P* < 0.05 and *P* < 0.01, respectively.

Gefitinib-based chemotherapy is considered the cornerstone in treating advanced lung cancer. To mimic long-time exposure of patients to gefitinib, an *in vitro* model was established by transforming human lung cancer PC9 cells via exposure to lower concentrations ofgefitinib for 24 weeks (Figure 2A). Interestingly, miR-30e amounts in PC9 cells wereelevated compared with values obtained in theresistant PC9Gcell line (Figure 2B). PC9G cells had resistancefeatures, including enhanced cell proliferation andmigration, alongside lower apoptosis rates (Figure 2C–2E). In addition, we found that PC9G showedincreased cell proliferation andmigration, with reduced apoptosis compared with PC9 cells.

### High miR-30e levels in PC9G cells inhibit cancer aggressiveness and reverses drug resistance to gefitinib

**Figure 3:**
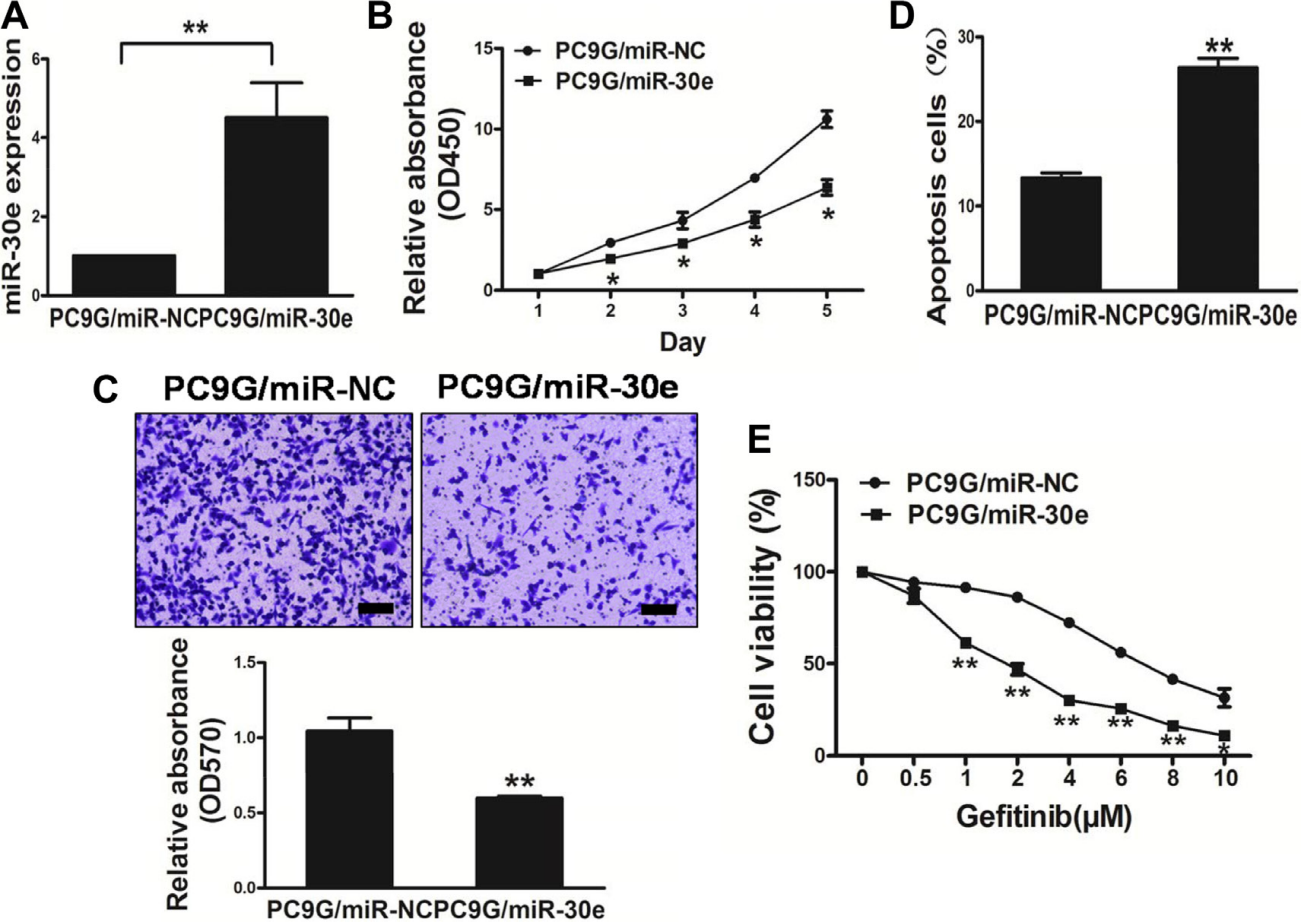
MicroRNA-30e overexpression in the PC9G cell linereduces cell proliferation andmigration, and reverses drug resistance to gefitinib (**A**) Real-time PCR quantifying miR-30e amounts in PC9G cells. (**B**) The CCK8 assay was used to quantitate cell viability after transduction with miR-30e or miR-NC. (**C**) Transwell migration assays were conducted for respective cells. (**D**) Apoptosis Assay was carried outfor respective cells. (**E**) Gefitinib sensitivity of the PC9G/miR-NC and PC9G/miR-30e cell lines was tested by CCK-8 assay. Data are mean ± SD of 3 replicate experiments. * and ** indicate significant differences at *P* < 0.05 and *P* < 0.01, respectively.

MiR-30e amounts in resistant PC9G cells were lower thanin PC9 cells. Interestingly, cell growth was reduced in miR-30e-overexpressing lung cancer cells in comparison with those transfected with miR-NC (Figure 3A and 3B). We next assessed the impact of miR-30e on cell migration. As shown in Figure 3C, miR-30e re-expression starkly reduced the migrationability of lung cancer cells. What's more, overexpressionof miR-30e promoted cell apoptosis (Figure 3D). We further found that miR-30eoverexpression reversed drug resistance to gefitinib in PC9G cells (Figure 3E). Thus, our results suggest miR-30e inhibited tumor aggressiveness, and reversed drug resistance to gefitinib.

### Repression of miR-30e in PC9 cells significantly promotes cell growth and migration, also conferring resistance to gefitinib

**Figure 4:**
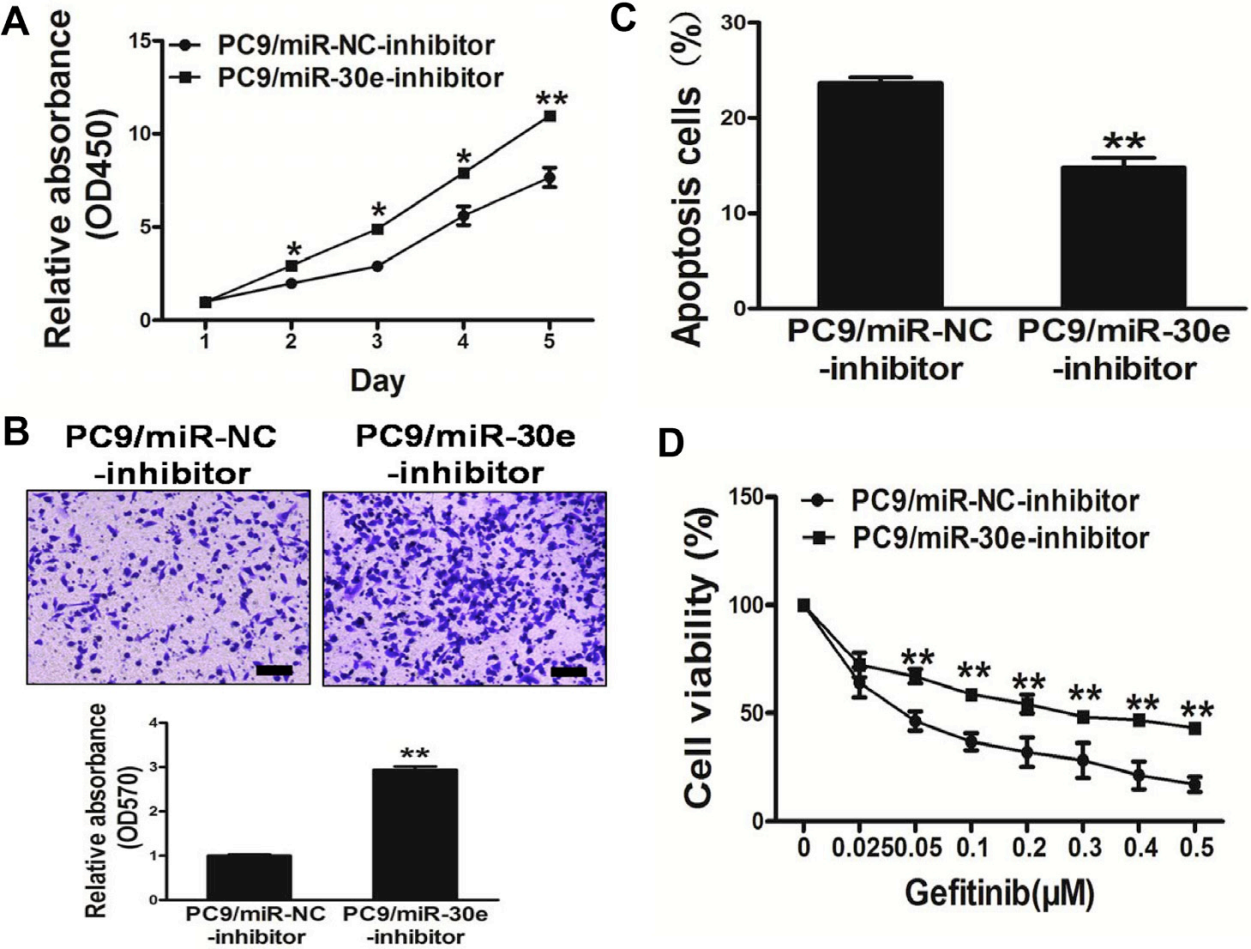
Repression of miR-30e in PC9 cells significantlypromotes cell growth and migration, and confers resistance to gefitinib (**A**) The CCK8 assay of PC9 cellswas performed after transduction with miR-30e-inhibitor or miR-NC-inhibitor. (**B**) Transwell migration assays werecarried out for respective cells. (**C**) Apoptosis assay was performed in respective cells. (**D**) Gefitinib sensitivity in PC9/miR-NC-inhibitor and PC9/miR-30e-inhibitor cell lines was tested by the CCK-8 assay. Data are mean ± SD of 3 replicate experiments. * and ** indicate significant differences at *P* < 0.05 and *P* < 0.01, respectively.

To evaluate miR-30e function in lung cancer carcinogenesis, PC9 cell growthwas assessed after transfection with miR-30e-inhibitor. Interestingly, markedly decreased miR-30e levels were observed after silencing of the miRNA, resulting in increased PC9 cell growth compared with the miR-NC-inhibitorgroup (Figure 4A).

Since migration is a very important malignancy feature, the effects ofmiR-30e on cellmigration was evaluated. As shown in Figure 4B, miR-30e-inhibition dramatically induced the normally strong migration capacity of lung cancer cells, promoting cell survival by inducing apoptosis (Figure 4C). Furthermore, inhibition of miR-30econferred resistance to gefitinib in PC9 cells (Figure 4D). Thus, these results suggest that repression of miR-30e in PC9 cells significantly promoted cell growth andmigration, while conferring resistance to gefitinib.

### MiR-30e sensitizes HCC827/GR cells to gefitinib

**Figure 5:**
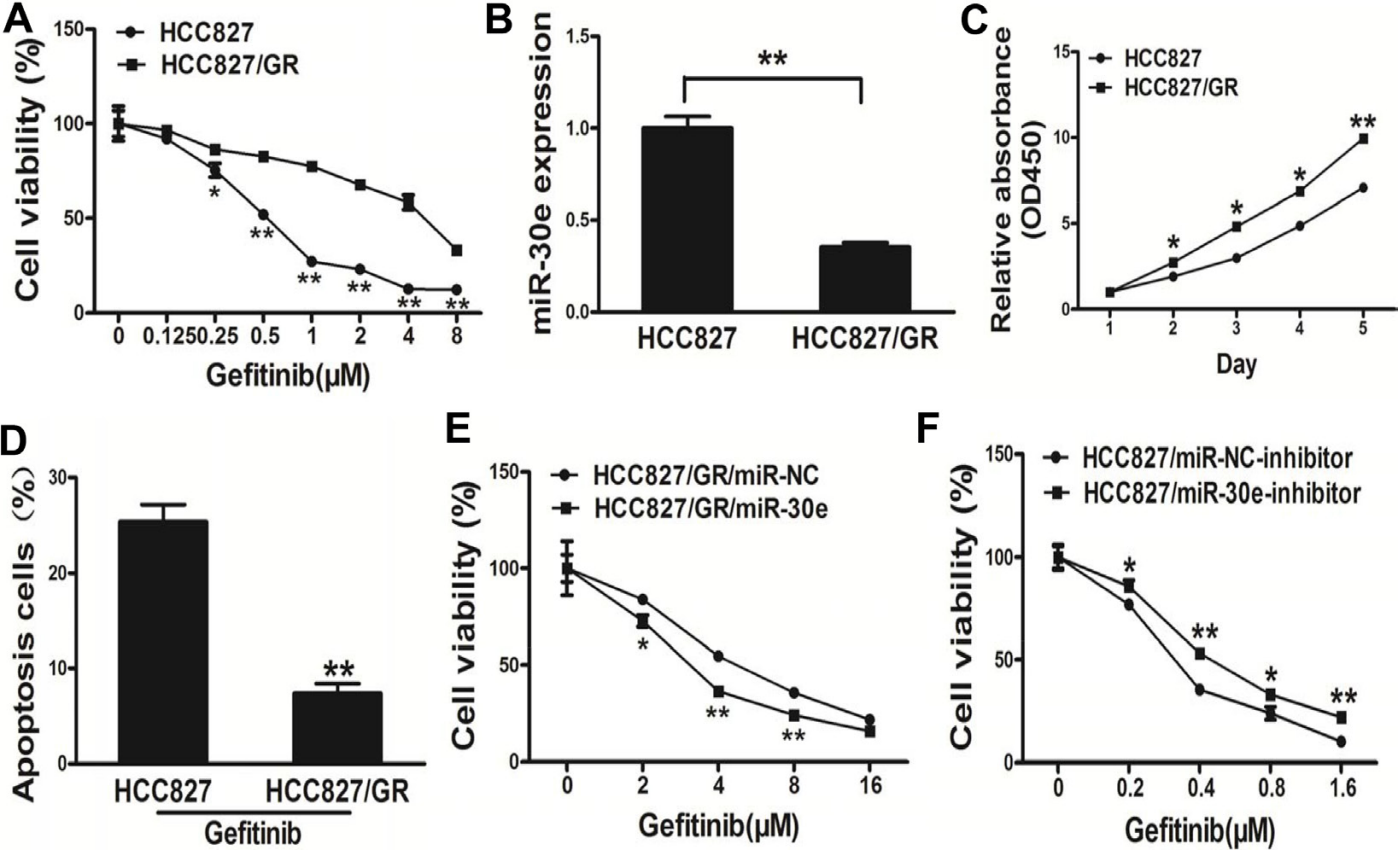
MicroRNA-30e renders HCC827/GR cells more sensitive to gefitinib (**A**) In comparison with the HCC827 cell line, HCC827/GR cells displayed less sensitivity to gefitinib. (**B**) MicroRNA-30e amounts in HCC827 and resistant HCC827/GR cells. (**C**) Apoptosis Assay were conducted in HCC827 andHCC827/GR cells. (**D**) The CCK8 assay of HCC827 andHCC827/GR cells was carried out at various time points. (**E**) Gefitinib sensitivity in HCC827/GR/miR-NC and HCC827/GR/miR-30e cell lines evaluated by the CCK-8 assay. (**F**) Gefitinib sensitivity in HCC827/miR-NC-inhibitor and HCC827 /miR-30e-inhibitor cell lines assessed by the CCK-8 assay.* and ** indicate significant differences at *P* < 0.05 and *P* < 0.01, respectively.

Next, we adopted thehuman lung cancer cellline HCC827 with its variant HCC827/GR cells which exposure to indicated lower concentration gefitinib for 24 weeks (Figure 5A). Interestingly, miR-30e amounts in HCC827 cells wereincreased compare with the values of the resistant HCC827/GR cells (Figure 5B). Meanwhile, HCC827/GR cells had resistance features, including elevated cell proliferation and a reduced apoptosis rate (Figure 5C–5D). Furthermore, high miR-30e amounts reversed drug resistance to gefitinib in HCC827/GR cells (Figure 5E). Meanwhile, inhibition of miR-30epromotedchemoresistance to gefitinib in HCC827 cell lines (Figure 5F). Our results suggested that miR-30e sensitized HCC827/GR cells to gefitinib.

### HOXA1 is a direct target of miR-30e

**Figure 6:**
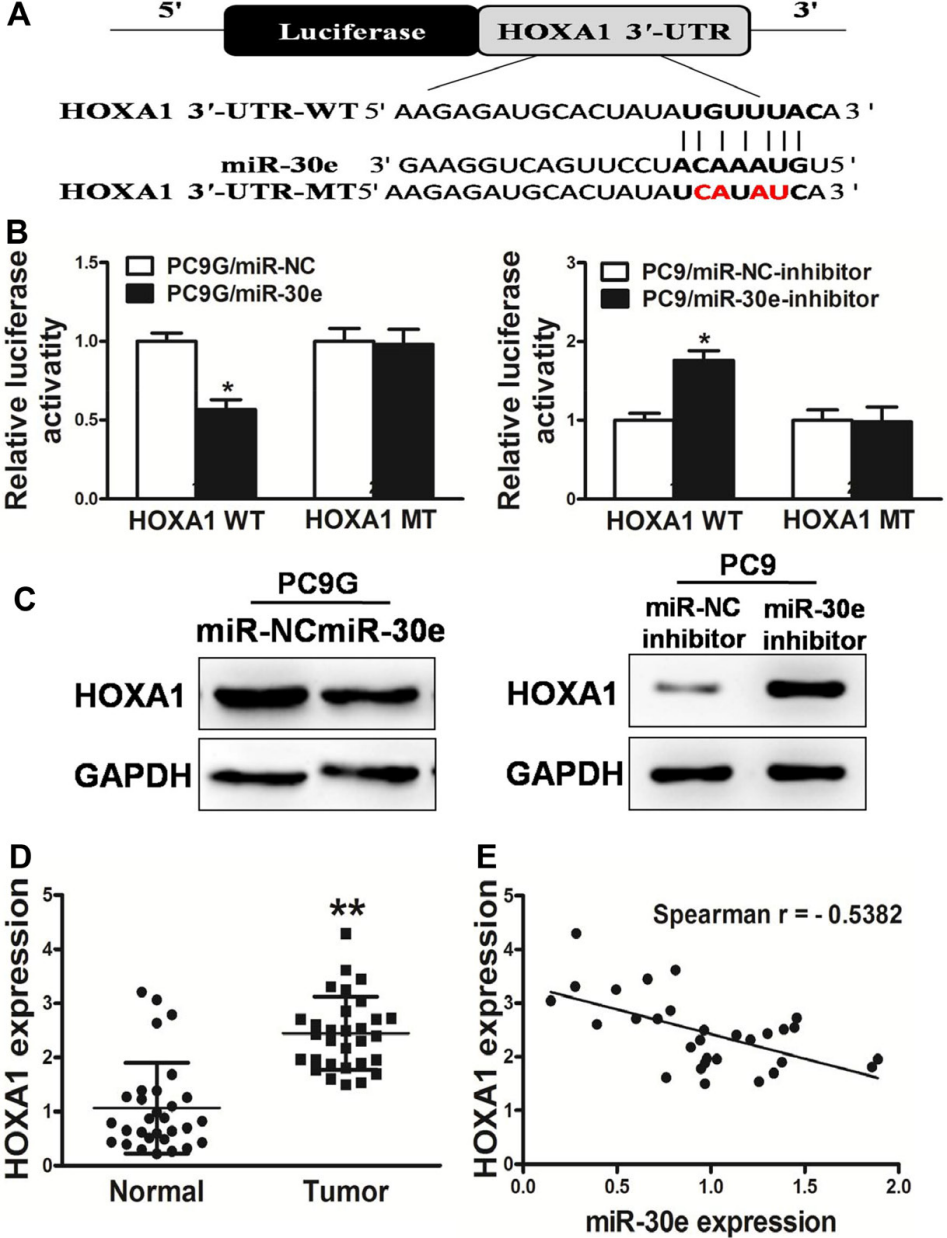
HOXA1 is a miR-30e target (**A**) MicroRNA-30e binding site in the human HOXA1 3′-UTR and a reporter construct depicting the whole HOXA1 3′-UTR fragment as well as the mutant HOXA1 3′-UTR (mutated nucleotides are shown in red). (**B**) Luciferase assay on PC9G or PC9 cells, co-transfected with mimics or inhibitor and a luciferase reporter comprising full length HOXA1 3′-UTR (WT) or mutated (MT) sequence with 4 changed nucleotides in the miR-30e binding site. Luciferase activitywas assessed 24 h after transfection. MicroRNA-30e starklyreduced luciferase activity. Data are mean ± SD (*n* = 4). (**C**) The expression of HOXA1 in cells was determined by western blotting analysis. (**D**) HOXA1 amounts in non-cancerous tissues and human lung cancersamples were assessed by RT-qPCR; fold changes were derived for HOXA1based on GAPDH amounts. (**E**) The association of HOXA1 amounts and miR-30elevels was determined by Spearman′s correlation. Data are mean ± SD of three replicate experiments. * and ** indicate significant differences at *P* < 0.05 and *P* < 0.01, respectively.

To explore the underlying mechanism of miR-30e in lung cancer, the database TargetScan (www.targetscan.org) was searched. We found that miR-30e likely regulates the HOXA1 gene sinceits 3′-UTR harbored the binding site for the seed region of miR-30e.HOXA1 has a critical function in normal tissue growth and differentiation. Based on the putative binding site of miR-30e in the 3′UTR of the HOXA1 gene, we initially constructed two types of plasmids containingthe luciferase reporting gene with wild-type or mutantHOXA1 3′UTR, and co-transfected miR-30e mimics or inhibitorinto PC9G or PC9 cells; interestingly, cells co-transfected withmiR-30e mimics and wild-type HOXA1 3′UTR showeda significant decrease in luciferase activity, whilemiR-30e-inhibitor significantlyincreasedthe luciferase activity. However, in the mutant group, no detectablechange in luciferase activity was observed (Figure 6A–6B), suggesting that miR-30e suppressed thetranscription activity of the HOXA1 gene by targeting the putative3′UTR of HOXA1 mRNA independently. Western blot demonstrated that HOXA1 protein amounts were reduced in miR-30e treated PC9G cells, and increasedafter miR-30e-inhibition in PC9 cells (Figure 6C). Furthermore, HOXA1amounts were assessed in human lung cancersamples and adjacent normal tissue specimens, with markedly increased values found in cancer specimens (Figure 6D). Next, the association ofHOXA1content with miR-30e amounts in human lung cancer specimens was assessed by Spearman's rank correlation. Interestingly, HOXA1content and miR-30e levels were inversely correlated in human lung cancer specimens (Spearman's correlation *r* = −0.5382) (Figure 6E). These data demonstrated that miR-30e directly targeted HOXA1 in lung cancer cells.

### MiR-30e enhances the chemosensitivity of gefitinib *in vivo*

**Figure 7:**
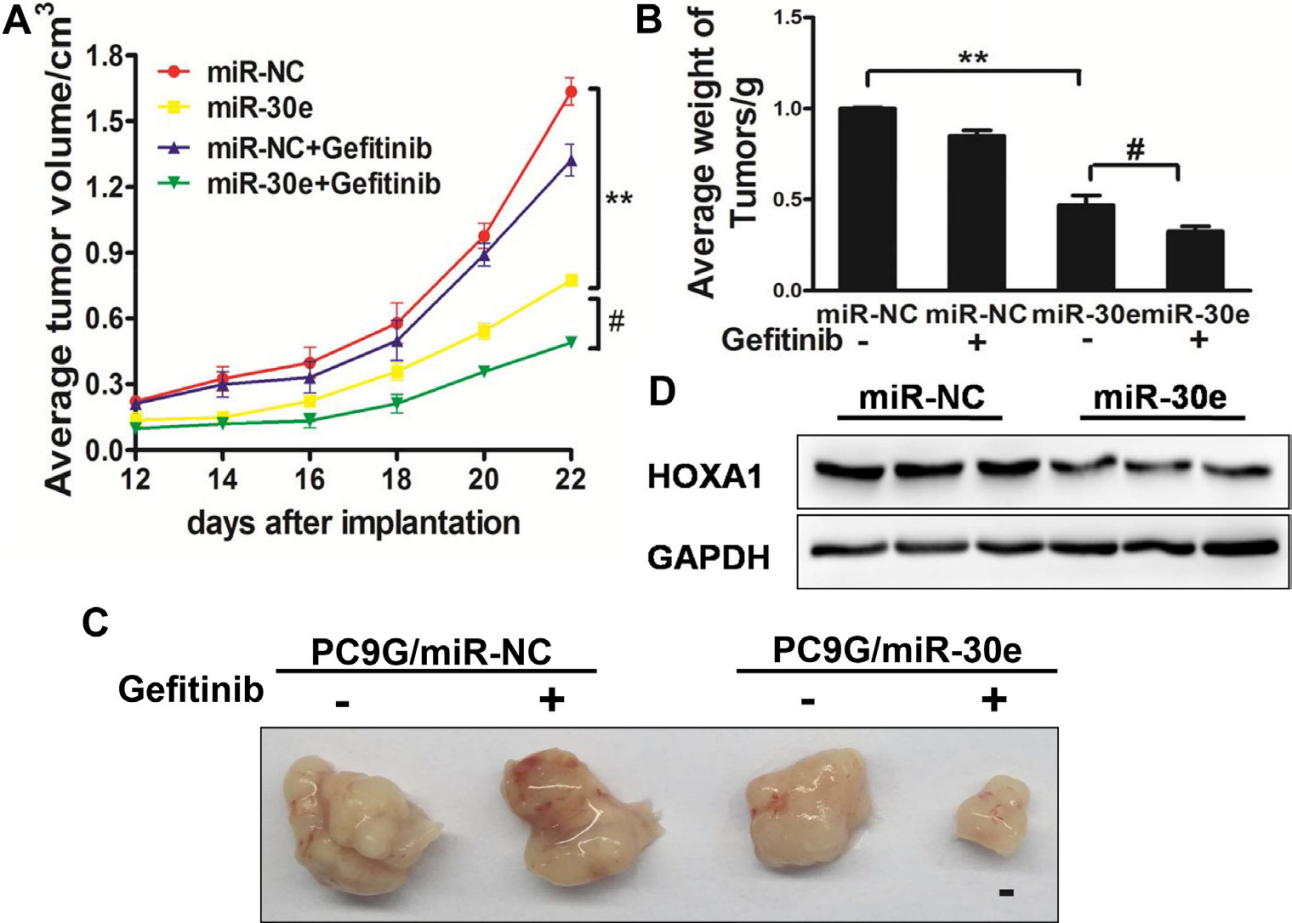
MiR-30e enhances chemosensitivitytogefitinib in a mouse model (**A**–**C**) MicroRNA-30e affectsPC9G cell growth in nude mice. BALB/c nude mice were subcutaneously administered 5 × 10^6^ cells transduced with lentiviruses carrying miR-NC or miR-30e. The tumorswere measured at different time points; gefitinibtreatment was carried out intraperitoneally. The tumorswere extracted and weighed at 24 days. MicroRNA-30e treatment resulted in decreased tumor volumes and weights compared with the miR-NC group; meanwhile, miR-30e plus gefitinibfurther inhibited tumor growth compared with the miR-30e group. Bar = 1 mm. (**D**) HOXA1amounts were assessed in tumor tissue specimens by immunoblotting. Data are mean ± SD. * and # indicate significant differences at *P* < 0.01 and *P* < 0.05, respectively.

To evaluate the function of miR-30e in canceraggressiveness *in vivo*, PC9G/miR-NC and PC9G/miR-30e cells were subcutaneously administered into both posterior flanks of male BALB/c nude mice. The formed tumors were measured every other day;gefitinib was administered by peritoneal injection. Interestingly, MiR-30e decreased tumor volumes and weights compared with the miR-NC group, and miR-30e plus gefitinibresulted in decreased tumor volumes and weights compared with miR-30e (Figure 7A–7C). To explore the molecular mechanisms by which miR-30e affects tumor growth, total protein samples were obtained for Western blot; as expected,HOXA1 protein amounts were reduced in miR-30eexpressing tumors (Figure 7D). These findings suggested that miR-30e enhanced lung cancer sensitivity to gefitinib in nude mice.

## DISCUSSION

MicroRNAs have significant functions in carcinogenesis, with some correlated with clinical characteristics and outcomes [22]. Meanwhile, lung cancertumorigenesis involves both genetic and epigenetic alterations, including the induction of oncogenes and/or suppression of tumor suppressors. Altered miRNA expression is commonly found in human carcinomas, e.g. NSCLC [23, 24]. Here, the role of miR-30e in lung carcinoma was evaluated, as well as the underlying molecular mechanisms.

Mounting evidence suggests that miR-30e is a potential tumor suppressor in multiple cancers. As shown above, reduced miR-30e levelswere obtained in lung cancer specimens, in comparison with adjacent non-cancerous tissue samples. The expression of miR-30e was reduced in the resistantlung carcinoma PC9G cell line in comparison with PC9 cells. In agreement, miR-30e overexpression resulted in decreased cell growth and migration, while inducingapoptosis in PC9G cells; conversely miR-30e repression markedly promoted cell growth and migration, and inhibited apoptosis in PC9 cells. This study is the first to reveal miR-30e overexpression in chemosensitivity. These findings could help developnovel therapeutic strategies for lung carcinoma treatment.

HOXA1, a member of the HOXA family which wasfirst identified in Drosophila, has been previously reported to significantly influence the normal growth and differentiationof mammalian tissues [25, 26]. HOXA1 re-expression in human mammary epithelial cells was shown to cause oncogenic transformation and tumorigenesis *in vivo* [27]. HOXA1 mutation results in decreased mammary cell proliferation, anchorageindependentgrowth, and loss of contact inhibition [28]. In addition, HOXA1 expression levels in squamous cell lung-and cervical cancer tissue samples are significantly elevated compared with adjacent normal tissue specimens [19, 29]. Here, the HOXA1 oncogene was further identified as anew miR-30e target both *in vitro* and in mice. First, luciferase reporter assay demonstrated that miR-30e directly recognized the 3′-UTR of HOXA1 mRNA. In addition, HOXA1 levels were significantly reduced after stable miR-30e expression. Thirdly, HOXA1 and miR-30ewere inversely correlated in clinical specimens. Taken together, these findings indicated that HOXA1 is anew miR-30e target.

MicroRNAs are considered to be involved in cancer chemoresistance; indeed, they are differentially expressed in chemo-sensitive and chemoresistant cells [30–32]. Interestingly, ectopic miR-34a was shown to sensitizecolorectal carcinoma cells to 5-fluorouracil [33]; meanwhile,miR-497 reduces tumor cell proliferation and sensitizes to 5-fluorouracil by inactivating KSR1 [34], and targets N-RAS to increase temozolomide-dependent apoptosis in gliomas [35]. As shown above,miR-30e enhances chemosensitivity togefitinib, both *in vitro* and *in vivo*, confirmingthatmiR-30e re-expression may constitute a novel strategy forovercoming chemoresistance to gefitinib in lung cancer. However, the majority of clinical samples did not undergoEGFR mutationassessmentbecause most patients were administeredgefitinib after unsuccessful chemotherapy and diseaseprogression. Thisis alimitation if this study. Therefore, it remains unclear whether theobserved response may be due to EGFR-mutations. We then retrospectively assessed samples with EGFR-mutation, which also showedelevatedmiR-30e amounts. These findings indicated that elevated miR-30e amounts may overlap to some extentwith EGFR-mutation, which is a very well-known predictive marker of response to EGFR-TKIs. Further research is required to testthis hypothesis.

In summary, the current findingsfirstly demonstrated that miR-30e played a significant role in suppressing lung cancer cell growth through HOXA1inhibition. Although we confirmed that miR-30e could inhibit lung cancer by targeting HOXA1, there might be other miR-30etargets, which could also affect tumor growth in lung cancer. Therefore, further studies are warranted for identifying additional targets and pathways modulated by miR-30e. Although the mechanisms underpinning lung cancer are currently more understood, treating this malignancy still constitutes a clinical challenge.

## MATERIALS AND METHODS

### Cell culture and clinical tissue specimens

Human lung cancer PC9, PC9G, HCC827 and HCC827/GR cells were maintained in RPMI 1640;the HEK-293T cell line was cultured in DMEM containing 10% fetal bovine serum (FBS), 100 IU/mL penicillinand 100 mg/mL streptomycin. Cell culture was carried out at 37°C in a humid atmosphere with 5% CO2. PC-9 and HCC-827 cellsharborthe activating EGFRmutation del E746-A750 in exon 19.

Lung cancer tissue samples and adjacent non-canceroustissue specimens were obtained from patients undergoing lung cancer resection, and snap-frozen in liquid nitrogen post-surgery.

### Lentiviral packaging of miR-30e and stable cell line establishment

A lentiviral packagingkit was usedtostably overexpress miR-30e in lung cancer cells. Lentivirus carryingmiR-30e or negative control (miR-NC) was packaged according to the manufacturer's instructions, in HEK-293T cells using polybrene (Sigma-Aldrich); selection was performed by treatment with puromycin (Sigma-Aldrich) for 2 weeks, to yield stablecell lines.

### RNA purification and real-time reverse transcription polymerase chain reaction (qRT-PCR)

Total RNA was obtained from cultured cells with TRIzol reagent (Invitrogen, USA) as instructed by the manufacturer. Quantitative real-time RT-PCR detecting mature miR-30e was carried out in triplicate with RT Reagent Kit (Vazyme, Nanjing, China) as directed by the manufacturer, withAceQ SYBR Master Mix (Vazyme, Nanjing, China) on a 7900HT system. MicroRNA-30e levels in each group were determined relative to U6 amounts, by the 2^−ΔΔCt^) method.

### Cell proliferation assay

Cell counting Kit-8 (CCK8 kit, Dojindo Laboratories, Japan) assay was used for cell viability assessment. A total of 2,000 cells were plated per well in 96-well plates, and cultured as described above for 48 h after transfection. After incubationforindicated times, CCK-8 reagent was supplemented per well and further incubated for 1–2 h. Absorbance was read at 450 nm.

### Migration assay

The effects of miR-30e on cell migrationwere investigated in 24-wellMatrigel invasion chambers (BD Biosciences, UK) as instructed by the manufacturer. Transfected cells (5 × 10^4^) were plated in upper wells in serum-free RPMI-1640, with RPMI-1640 containing 10% FBS in lower chambers. After 16-20 h, non-invading cells (top wells) were removed; invasive cells (bottom wells) were submitted to staining with 0.1% crystal violet after fixation (paraformaldehyde). Photomicrographs were captured in 3 randomly selected high power fields. After air drying, the membranes were treated with 33% acetic acid (300 μL/well) at room temperature for 15 minutes, and the resulting solutions transferred into 96-well plates. Absorbance at a wavelength of 570 nm was recorded.

### Western blotting

Cells were treated as described above for 48 h, and lysed in RIPA buffer containing protease inhibitorson ice for 30 min. Total protein amounts were assessed by the BCA assay (Beyotime, China). Equal amounts of protein were then separated by 10% SDS-PAGE. Subsequently, protein bands were electrically transferred onto nitrocellulose membranes (Whatman, Germany), which were incubated with anti-HOXA1 (Proteintech Technology, USA) and anti-GAPDH (Bioworld Technology, USA) antibodies at 4°C overnight.

### Luciferase reporter assay

TargetScan was employed to predict miR-30e binding sites. A fragment of the 3′-UTR of HOXA1 with the putative miR-30e binding site was cloned by PCR. To generate a construct harboring mutated miR-30e binding site, four nucleotides corresponding to the 5′-seeding region of this site were substituted in the wild type fragment. The complementary fragment in the 3′-UTR of HOXA1 (UGUUUAC) was mutated to UCAUAUC. PCR products were cut with SacI and HindIII, cloned inserted into pMIR-REPORTER, and validated by DNA sequencing. Constructs were then co-transfected with miR-30e or miR-NC into HEK-293 cells in 24-well plates for 24 h, followed by Luciferase assays with Dual Luciferase Reporter Assay System (Promega, WI, USA).

### Apoptosis assay

Apoptosis was assessed flow-cytometrically, afterstaining with AnnexinV and propidium iodide (BD Pharmingen) according to the manufacturer's instructions. Analysis was carried out on FACSCanto II (BD Biosciences) with the FlowJo software.

### Xenograft studies

BALB/cA-nu (nu/nu) nude mice (male, 6-weeks old], purchased from Shanghai Laboratory Animal Center (Chinese Academy of Sciences, Shanghai, China), were housed in a specific pathogen-free (SPF) vivarium. Aliquots of cells (5 × 10^6^) in 150 mL FBS-free RPMI 1640 was subcutaneously administered into both posterior flanks of the animals. Tumors were measured withVernier calipers every 2 days, with volumesderived as followed: Volume = 0.5 × Length × Width^2^. Ten days after implantation, gefitinib (5 μM) was intraperitoneal injected in the indicated mice. The animals were euthanized 24 days after implantation, extracting the tumors.

### Statistical analysis

Experiments were carried out in triplicate, with data assessed using GraphPad Prism 5 (La Jolla, CA, USA). The association of miR-30e levels with HOXA1 amounts in human lung cancer wasassessed by Spearman's rank test. Group comparison was carried out by *t-test*. Statistical significance level was set at *P* < 0.05.
